# Course and outcome of comorbid mood disorders in children and adolescents with intellectual disability [IDD]

**DOI:** 10.1192/bjo.2021.694

**Published:** 2021-06-18

**Authors:** Arul Jayendra Pradeep Velusamy, Satish Chandra Girimaji, John Vijay Sagar Kommu

**Affiliations:** 1 Former Senior resident, National Institute of Mental Health and Neuro Sciences [NIMHANS], RCPsych MTI Trainee, Central and North West London NHS Trust; 2 Professor, Department of Child and Adolescent Psychiatry, National Institute of Mental health and Neuro Sciences [NIMHANS]

## Abstract

**Aims:**

The primary objective of the study is to assess the clinical course and functional outcome of comorbid mood disorders in children and adolescents with Intellectual Disability [IDD]

**Method:**

53 children and adolescents with varying levels of severity of IDD presenting with comorbid mood disorders diagnosed using Kiddie Schedule for Affective Disorder and Schizophrenia-DSM-5 version [KSADS] were recruited by convenient sampling with the exclusion of autism spectrum disorders. Vineland Social Maturity Scale [VSMS] is used to quantify the severity of ID. Developmental Behaviour Checklist-Parent [DBC-P] version is used to measure psychopathology, Clinical Global severity of illness [CGI-S] to quantify the clinical improvement, and Developmental Disabilities Children's Global Assessment of Severity [DD-CGAS] to assess functional improvement. Prospective naturalistic follow-up was done with assessment points at baseline, 1, 3- and 6-month timeline.

**Result:**

40 patients were followed up for 6 months period. Overall significant improvement is observed in the dependent variables like CGI, DDCGAS, and DBC-P from baseline to 3 months and then a plateau of improvement from 3 to 6 months. The diagnostic breakup of mood disorders is mania [N = 19], Depression [N = 12], and mixed affective state [N = 9]. Patients with mania had significant improvement in DBC score [F = 12.69, p < 0.001 in repeated measures ANOVA], DDCGAS [p < 0.001], and CGI score [p < 0.03] with an overall remission rate of 42.10% over 6 months period. Patients with depression had significant improvement in DBC score [F = 15.48, p < 0.001], DDCGAS, and CGI score [p < 0.001] with an overall remission rate of 41.7%. None of the patients with mixed affective states had clinical remission with no significant improvement observed in any of the dependent variables measuring course and outcome.

**Conclusion:**

Comorbid psychiatric disorders in children and adolescents with IDD have a guarded prognosis compared to mood disorders in neurotypical children. Comorbid ADHD and caregiver stress majorly influenced the course and outcome in the current study.

